# Neonatal sepsis and its associated factors among neonates admitted to the neonatal intensive care unit in Wachemo University Comprehensive Specialized Hospital, Southern Ethiopia, 2022

**DOI:** 10.3389/fped.2023.1184205

**Published:** 2023-07-03

**Authors:** Taye Mezgebu, Getachew Ossabo, Asnakech Zekiwos, Hamdino Mohammed, Zerihun Demisse

**Affiliations:** ^1^ Department of Comprehensive Nursing, Schools of Nursing, College of Health Science and Medicine, Wachemo University, Hosanna, Ethiopia; ^2^ Department of Pediatrics and Child Health Nursing, Schools of Nursing, College of Health Science and Medicine, Wachemo University, Hosanna, Ethiopia

**Keywords:** neonate, neonatal sepsis, intensive care unit, Ethiopia, NICU (neonatal intensive care unit)

## Abstract

**Background:**

Neonatal sepsis is a major public health problem worldwide. It is one of the leading causes of neonatal mortality and morbidity worldwide. The neonatal mortality rate is higher in developing countries, where the extent and causes of neonatal sepsis are not yet known. Neonatal sepsis is a leading cause of neonatal mortality in Ethiopia. As a result, this study aimed to assess the proportion and identify maternal and neonatal risk factors for neonatal sepsis among neonates admitted to the neonatal intensive care unit.

**Methods:**

An institutional-based cross-sectional study was conducted from May 2022 to July 2022 at the Wachemo University Comprehensive Specialized Teaching Hospital, Neonatal Intensive Care Unit, southern Ethiopia. A total of 205 neonates with indexed mothers participated in the study. Using a consecutive sampling technique, a structured, pretested questionnaire was used to collect data from the study subjects. Data were entered into EpiData Manager version 3.1 for Windows and then exported to SPSS version 22 for further data cleaning and analysis. Descriptive analyses were performed by using frequency, percentage, and summary statistics to describe the key variables. A multivariate regression model was used to identify factors associated with neonatal sepsis. Finally, statistical significance was declared at a *p*-value of less than 0.05, and an adjusted odds ratio (AOR) with a 95% confidence level was used to declare the variable’s association with the outcome variable.

**Result:**

The overall prevalence rate of neonatal sepsis was 39.5% (95% CI: 33.7–45.9). Multivariable analysis was performed by taking a variable that is statistically significant in bivariate logistic regression as a candidate variable. Multivariable model analysis showed that unmarried status AOR = 18.37 (95% CI: 1.56–216.14), maternal fever during delivery AOR = 4.74 (95% CI: 1.63–13.8), and premature rupture of membrane AOR = 7.53 (95% CI: 2.19–25.6) were variables that increased the odds of developing neonatal sepsis.

**Conclusion:**

The study’s findings indicate that neonatal sepsis is highly prevalent. Unmarried maternal status, maternal fever during delivery, and premature rupture of the membrane were predictors of neonatal sepsis. Therefore, providing training for health workers and close monitoring and evaluation during obstetric and neonatal care are crucial to halt the occurrence of neonatal sepsis.

## Introduction

The neonatal period starts at birth and ends after 28 days of life. As a result of birth and labor insult, neonates may encounter health challenges. Neonatal sepsis (NNS) is defined as a systemic inflammatory response syndrome that ranges from subclinical infection to severe or systemic manifestation caused by bacteria, viruses, or fungi in a neonate (1–4). It is categorized into two types: early-onset neonatal sepsis (EONS), which occurs within the first 7 days of life due to microorganisms and is transmitted vertically from mother to infant, and late-onset neonatal sepsis (LONS), where sepsis occurs in neonatal intensive care unit (NICU) infants from the 8th to 28th day of life (3, 5, 6).

Globally, neonatal sepsis is one of the major public health problems, and it is a significant cause of morbidity and mortality during the neonatal period. Surprisingly, it is the second leading cause of death in neonates, accounting for 24% of deaths (7, 8). According to epidemiological estimates, there were 1.7 million cases of neonatal sepsis worldwide (9). According to the World Health Organization, neonatal deaths account for nearly 45% of all under-five deaths. Out of 5.9 million child deaths in 2015, almost 1 million occurred on the first day of life, whereas nearly 2 million occurred in the first week (10). A systematic and meta-analysis study showed that the magnitude of newborn mortality is higher in low-income countries such as sub-Saharan Africa, South Asia, and Latin America. Substantially, 99% of deaths occur in low- and middle-income countries (11).

The risk of neonatal death in developing countries has higher burden than that in developed countries (12). Likewise, compared with high-income countries, neonatal sepsis is approximately 40 times more prevalent in middle-income countries, and its mortality rate is nearly two times higher (13, 14). Its prevalence and mortality also varied substantially across regions of the world, with the highest burden in sub-Saharan Africa, Oceania, South Asia, East Asia, and Southeast Asia. In Africa, sepsis constitutes 28% of all neonatal deaths (15). Apart from this, the prevalence of NNS varies from country to country throughout Africa. For instance, the prevalence rates are 37.9%, 37.6%, 34.1%, and 29.3% in Cameroon, Nigeria (16), Tanzania (17), and Kenya (18), respectively.

According to the 2019 Ethiopian Mini Demographic Health Survey, neonatal, infant, and under-five mortality rates were 33, 47, and 59 deaths per 1,000 live births, respectively. In other words, 1 in every 30 Ethiopian children dies within the first month, 1 in every 17 dies before their fifth birthday, and 1 in every 21 dies before their first birth date (19). Despite the advancement of service delivery facilities and management approaches in different parts of Ethiopia, the prevalence of NNS also varied. For instance, a high prevalence rate was reported in northeast Ethiopia, 79.4% (20); Arba Minch Hospital, 78.3% (21, 22); and Shashamane, 77.9% (23). In addition, its prevalence rate was 64.8%, 52.6%, 52.3%, and 45.8% in central Gondar, Jimma, northern Oromia hospitals, and eastern Ethiopia hospitals, respectively (24–26).

Apart from the high prevalence rate of NNS, different maternal and neonatal risk factors play a significant role in the development of neonatal sepsis, such as birth asphyxia, low birth weight, low socio-economic status, prematurity, surgical procedure, congenital malformations, complex or instrument-assisted delivery, gestational age, parity, cesarean delivery, urinary tract infection in the third trimester of pregnancy, meconium-stained amniotic fluid, and place of delivery (3, 10, 16, 24, 27–30).

Early identification of neonatal sepsis risk factors can reduce the likelihood of infant mortality and morbidity. In addition to unspecified symptoms and signs, lack of skilled personnel, and resource-limited facilities, neonatal sepsis case identification and care are particularly challenging in Ethiopia. Hence, identifying neonatal sepsis risk factors can reduce the likelihood of infant mortality and morbidity. The impact of neonatal sepsis remains a major public health problem in resource-limited settings such as Ethiopia, where there are scant findings on the magnitude and risk factors for neonatal sepsis; also, no study was previously done in our study area. Therefore, to achieve the Sustainable Development Goal, which aims to end preventable death of neonates and children under five years of age, maintaining skilled as well as clean delivery, scaling up maternal immunization, and securing clean umbilical cord cutting, early detection and treatment, and closed medication systems are expected approaches to prevent NNS (31, 32).

Without the reduction of direct causes of NNS, the desired goal may not be attained. Thus, the purpose of this study was to determine the prevalence and contributing factors of neonatal sepsis among newborns hospitalized in the intensive care unit of Wachemo University Comprehensive Specialty Teaching Hospital in southern Ethiopia.

## Materials and methods

### Study area and period

A study was conducted at Wachemo University Nigist Eleni Mohammed Memorial Comprehensive Specialized Teaching Hospital (WCUNEMMCSTH), located in Hosanna Town, the capital city of the Hadiya Zone in southern Ethiopia. It is about 232 km from Addis Ababa, the capital city of Ethiopia. WCUNEMMCSTH is one of the largest hospitals in Ethiopia, with more than 2 million people in the entire Hadiya Zone and a part of the Kembata Tembero and Silite Zone as a referral hospital in the southern part of Ethiopia. The hospital has different departments and units. Pediatrics and child health is one department that encompasses the pediatric ward, emergency triage assessment and treatment, under-five outpatient department, and NICU. Within the NICU, there are 20 beds, 3 incubators, 2 radiant warmers, and 2 phototherapy units for newborn infants. It was one of the largest NICUs in the southern region of the state, with a very high patient admission rate. It also has a very high patient flow of 150 neonates per month on average.

### Study design and period

An institutional-based cross-section study was conducted from 1 May 2022 to 30 July 2022 at Wachemo University Comprehensive Specialized Hospital, southern Ethiopia.

### Population

The target population for the study was all neonates admitted to the NICU. In contrast, the study population included all neonates admitted to the NICU with indexed mothers who were selected randomly during the study period.

### Eligibility criteria

All neonates admitted to the NICU with different health problems and all mothers of neonates who volunteered to participate and were able to communicate were included in the study, whereas neonates who had congenital abnormalities, who were admitted with sepsis two or more times during the study period (to prevent double counting), and with incomplete patient chart information were excluded from the study.

### Sample size determination

Using the single population proportion formula, the sample size was calculated using the following assumptions: 95% confidence level (for the standard normal deviation with the corresponding value = 1.96) and a 5% margin of error, and taking the prevalence of neonatal sepsis at 52.3% from a previous study performed in northern Oromia (25). Since the total number of our study population (neonates admitted to the NICU within the past year) was less than 10,000, the final sample size estimated by using the correction formula was 205.

### Sampling technique and procedures

WCUNEMMCSTH was purposively selected because it is the only hospital with NICU in the zone, and it immensely renders service in the area. A systematic random sampling method was used to select the study participants among neonates admitted to the NICU. Ahead of daily quota allocation, a chart review (the total number of neonates admitted within NICU during the last two months was surveyed) and then an average daily sample for two months was determined. A total of 427 neonates were admitted to the NICU of WCUNEMMCSTH. This is the reason why our data collection period is 2 months. Based on this, our source population was 427. The total number (*N* = 427) of neonates admitted in the NICU within the 2-month period was divided by the minimum adjusted sample size (*n* = 205) to get a sampling interval which yields the *K*th value (*K* = 427/205) = 2, so every *K*th interval was considered to pick the study unit. The first number from the first two neonates with their mothers was selected using a lottery method. Finally, the study units were selected every two intervals until the desired sample size was achieved.

### Data collection tools and procedures

The tool was primarily prepared in English and then translated into the local language “Amharic” to ensure the clarity of questions for the respondents and better understanding, which was developed by reviewing different works of previous kinds of literature (17, 24, 25, 30, 33) used in this study. Before the actual data collection, a pretest was conducted in Durame General Hospital in the Kembata zone of the southern regional state by taking 15% of our sample size where no actual data collection took place. Based on the information obtained and testing compatibility with data entry software, a few amendments were made to the instrument accordingly. Before the actual date of data collection, data collectors were trained about the data collection procedures and how to handle the data, as well as the instrument content and participant confidentiality. Pretested interviewer-administered questionnaires and checklists were also used to collect the data from registration book records among sampled neonates admitted to the NICU. Four trained, well-experienced BSc nurses collected the data under the close supervision of principal investigators.

### Operational definition

**Neonatal sepsis:** Neonates were diagnosed as having neonatal sepsis if they presented with any of the following systemic manifestations: poor feeding, convulsions, drowsiness or unconsciousness, movement only when stimulated or no movement at all, fast breathing (greater than 60 breaths per minute), grunting, severe chest in-drawing, raised temperature > 38°C, hypothermia 35.5°C, central cyanosis or severe jaundice, severe abdominal distension, and local infection (24, 27, 34).

**Early-onset neonatal sepsis:** Neonates were diagnosed as having early-onset neonatal sepsis if neonatal sepsis develops before the age of 7 days of life (22).

**Late-onset neonatal sepsis:** Neonates were diagnosed as having late-onset neonatal sepsis if neonatal sepsis develops between the ages of 8 and 28 days of life (35).

### Data processing and analysis

All collected data were double-cross-checked for completeness. Then, all cleaned and coded data were entered into the EpiData version 3.1 software for Windows. Lastly, data were exported to SPSS version 20 for further cleaning, recording, and analysis. To describe the research variables concerning the population, descriptive statistics such as frequency, proportions, and percentages were generated and displayed in tables and graphs. To identify associated factors, bivariate and multivariate logistic regression analyses were performed. To determine the relationship between the two variables and to select candidate variables for the next model, bivariate logistic regression was performed. Model fitness was cross-checked by computing Hosmer–Lemeshow goodness of fit.

Controlling other confounding variables and adjusting for probable confounders, variables with a *p*-value of less than 0.25 were incorporated into a multivariable logistic regression model. Finally, variables with a *p*-value less than 0.05 at a 95% confidence interval were considered significantly associated with outcome variables by looking at an adjusted odds ratio (AOR).

### Ethical consideration

The Wachemo University institutional review board approved clinical clearance (ref = 915/14). Wachemo University wrote a formal cooperation letter and submitted it to Wachemo University Comprehensive Specialized Hospital Administrative to obtain their cooperation. The study purpose and confidentiality were explained to the study subjects. The study was conducted in accordance with the Declaration of Helsinki. At the time of data collection, verbal consent was obtained from the participants' mothers to confirm their willingness to participate. Those not willing to participate were given the right not to do so. Confidentiality of responses was also ensured throughout the research process.

## Result

### Socio-demographic characteristics of study participants

A total of 205 mother–neonate pairs participated in the study, with a 100% response rate. The mean age of the mothers of the neonates was 26.93 years (±0.45 SD). More than half of the mothers, 134 (65.5%), lived in rural areas. Nearly one-third were employees, and only 19% attended college and above (Table 1).

**Table 1:** Socio-demographic characteristics of indexed mothers of neonates admitted to the NICU of Wachemo University Comprehensive Specialized Hospital, southern Ethiopia, 2022.

Categorical variables		Frequency	Percent
Age per year	<20	24	11.7
	20–34	164	80.0
	≥35	17	8.3
Residence	Urban	71	34.6
	Rural	134	65.5
Marital status	Single	8	3.9
	Married	197	96.1
Occupation	Government employee	35	17.1
	Private employee	42	20.5
	Merchant	46	22.4
	Housewife	82	40.0
Educational status	Can’t read and write	25	12.2
	Literate	49	23.9
	Grades 1–8	58	28.3
	Grades 9–12	34	16.6
	College & above	39	19.0
Monthly income (Ethiopian Birr)	<900	15	7.3
	900–1,200	12	5.9
	1,201–2,050	32	15.6
	>2,050	146	71.2

### Obstetric and gynecologic characteristics of indexed mothers

Of all the mothers who participated, 109 (53.2%) were multipara. Of all the mothers who had antenatal care (ANC) visits, 117 (60.6%) had four or more visits. One-fourth of the mothers had a history of high-grade fever during delivery, and 26 (12.7%) of them confirmed having urinary tract infection. Of the majority, 197 (96.1) neonates born in health facilities, 164 (80.0%) of mothers gave birth with spontaneous vaginal delivery. However, 12 (5.9%) mothers had no ANC visits, and only 8 (3.9%) gave birth at home. While in labor, 65 (31.7%) mothers had >4 digital vaginal examinations, and 93 (45.4%) had meconium-stained amniotic fluid. In addition to current obstetric requirements, the mothers' history was also assessed. Likewise, 22 and 12 mothers have experienced pregnancy-induced disease and pregnancy-induced hypertension, respectively. Concerning the duration of premature rupture of membrane (PROM), 21 (10.3%) lasted for ≥18 h (Table 2).

**Table 2:** Obstetrics and gynecologic characteristics of indexed mothers at NICU, in Wachemo University Comprehensive Specialized Teaching Hospital, southern Ethiopia, 2022.

Categorical variables		Frequency	Percent
Parity	Primipara	96	46.8
	Multipara	109	53.2
Frequency of ANC visits	One to three times	76	39.4
	Four and above	117	60.6
History of high-grade fever	Yes	51	24.9
	No	154	75.1
Maternal UTI	Yes	26	12.7
	No	179	87.3
History of chorioamnionitis	Yes	43	21.0
	No	162	79.0
History of MSAF	Yes	93	45.4
	No	104	50.7
Types of pregnancy-induced diseases (PID)	PIH	12	5.9
	Anemia	7	3.4
	GDM	3	1.5
Place of delivery	Hospital	166	81.0
	Health center	31	15.1
	Home	8	3.9
History of PROM	Yes	77	37.6
	No	120	58.5
Duration of labor	<6 h	34	16.6
	6–12 h	103	50.2
	12–24 h	60	29.3
Frequency of DVE	≤3	132	64.4
	≥4	65	31.7

DVE, digital vaginal examination; PID, pelvic inflammatory diseases; GDM, gestational diabetes mellitus; ANC, antenatal care; MSAF, meconium-stained amniotic fluid.

### Neonatal characteristics

Neonates with ages ranging from 1 to 28 days, with a mean age of 4.6 days (SD + 6.77), were included in the study. More than half, 122 (59.5%), of the neonates were male, and 167 (81.5%) were in the age group of 0–7 days (early neonatal period). With regard to gestational age, 146 (71.2%) had a full term (37–42 weeks), and 57 (27.8%) and 2 (1%) had preterm and postterm, respectively. Nearly three-fourths (73.6%) of the neonates were born with normal birth weights, but almost one-fourth (24.4%) had low birth weights. One hundred fifty-seven (79.7%) have had a low appearance, pulse, grimace, activity, respiration (APGAR) score of ≤6, and 40 (20.3%) had an APGAR ≥ 7. Of the total neonatal study population, 124 (60.5%) experienced health problems, including meningitis, acute respiratory distress syndrome, and neonatal sepsis. The majority, 137 (66.8%), of the neonates developed birth asphyxia.

### Prevalence of neonatal sepsis

**Figure 1:**
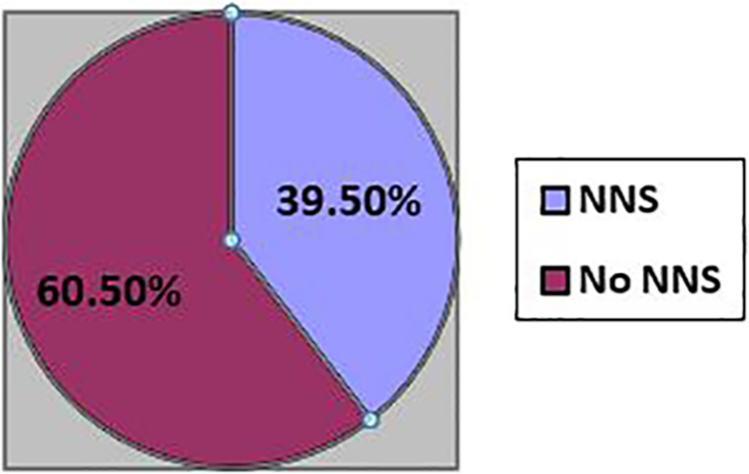
Prevalence of neonatal sepsis among neonates admitted with indexed women in NICU, WCUNEMMCSTH, southern Ethiopia.

The overall proportion of neonatal sepsis was 39.5% (95% CI: 33.7–45.9); the remaining 60.5% of the neonates were admitted with no sepsis. Among those who were admitted as of developing neonatal sepsis, 62 (76.5%) had early-onset neonatal sepsis, whereas 19 (23.5%) of them had late-onset neonatal sepsis (Figure 1: Prevalence of NNS).

### Factors associated with neonatal sepsis

The variables which fulfilled the chi-squared assumption were fitted and incorporated into the bivariate model to select the candidate variable for the multivariate model. Among background of neonate and index mother variables: age of mother, marital status, level of education, occupation of mother, sex, and maternal fever Neonatal variable: Chorioamnionitis, PROM, and duration of PROM, were statistically significant at *p*-value <0.25. Then, variables that were statistically significant in the bivariate logistic regression analysis were incorporated into multivariate analysis. Finally, determinant variables of neonatal sepsis were declared by looking at AOR at *p*-value < 0.05. Consequently, the unmarried status of mothers, maternal fever, and PROM were determinants of neonatal sepsis.

Accordingly, this study showed that neonates born from unmarried mothers had a significant association with the onset of neonatal sepsis. The odds of having neonatal sepsis among neonates born from unmarried mothers were 18 times higher than those among neonates born from married mothers [AOR = 18.37 (95% CI: 1.56–216.14)].

Maternal fever during delivery was found to be a significant risk factor for the development of NNS. Neonates born from mothers who experienced high-grade fever have an increased odds of development of neonatal sepsis by five folds [AOR = 4.74 (95% CI: 1.63–13.80)] as compared with those neonates born from mothers who did not have a fever during delivery. Neonates who were born from a mother who had a history of PROM before the onset of labor were seven times [AOR = 7.53 (95% CI: 2.19–25.6)] more likely to develop neonatal sepsis as compared with their counterparts (Table 3).

**Table 3:** Bivariate and multivariable analysis of factors associated with neonatal sepsis among neonates admitted to the NICU of WCUNEMMCSTH, southern Ethiopia, 2022.

Variables	Category	Neonatal sepsis		COR (95% CI)	AOR (95% CI)	*p*-value
		Yes	No			
Age	<20	17 (70.8%)	7 (29.2%)	4.5 (1.2–16.8)	1.163 (0.214–6.34)	0.86
	20–34	58 (35.4%)	106 (64.6%)	1.003 (0.35–2.85)	0.52 (0.143–1.89)	0.32
	≥35	6 (35.3%)	11 (64.7%)	1	1	
Marital status	Single	7 (87.5%)	1 (12.5%)	11.6 (1.404–96.454)	18.37 (1.56–216.1)	**0**.021**
	Married	74 (37.6%)	123 (62.4%)	1	1	
Educational status	Can’t read and write	9 (36.0%)	16 (64.0%)	1.875 (0.621–5.663)	1.12 (0.112–11.14)	0.924
	Read & write	20 (40.8%)	29 (59.2%)	2.299 (0.9–5.872)	2.32 (0.25–21.9)	0.46
	Grades 1–8	27 (46.6%)	31 (53.4%)	2.903 (1.173–7.185)	1.7 (0.19–15.7)	0.64
	Grades 9–12	16 (47.1%)	18 (52.9%)	2.963 (1.085–8.088)	1.193 (0.121–11.8)	0.88
	College & above	9 (23.1%)	30 (76.9%)	1	1	
Occupation	Gov., temp	8 (22.9%)	27 (77.1%)	1	1	
	Private employee	23 (54.8%)	19 (45.2%)	4.086 (1.509–11.06)	1.29 (0.13–12.8)	0.83
	Merchant	19 (41.3%)	27 (58.7%)	2.375 (0.89–6.35)	1.08 (0.109–10.73)	0.95
	Housewife	31 (37.8%)	51 (62.2%)	2.051 (0.83–5.08)	0.72 (0.07–7.3)	0.78
History of high-grade fever	Yes	40 (78.4%)	11 (21.6%)	10.2 (4.7–21.37)	4.74 (1.63–13.8)	**0**.004**
	No	41 (26.6%)	113 (73.4%)	1	1	
Chorioamnionitis	Yes	33 (76.7%)	10 (23.3%)	7.84 (3.58–17.16)	2.37 (0.71–7.9	0.16
	No	48 (29.6%)	114 (70.4%)	1	1	
PROM	Yes	46 (59.7%)	31 (40.3%)	4.5 (2.4–8.23)	7.53 (2.19–25.96)	**0**.01**
	No	30 (25%)	90 (75%)	1	1	
Duration of PROM	<18 h	31 (55.4%)	25 (44.6%)	1	1	
	≥18 h	15 (71.4%)	6 (28.6%)	6.64 (2.4–18.5)	0.31 (0.084–1.16)	0.083
Sex of neonates	Male	55 (45.1%)	67 (54.9%)	1.8 (1.003–3.23)	1.5 (0.73–3.3)	0.25
	Female	26 (31.3%)	57 (68.7%)	1	1	

PROM, premature rupture of membrane; AOR, adjusted odds ratio; COR, crude odds ratio; NICU, neonatal intensive care unit.

**Statistically significant at *p*-value < 0.05.

## Discussion

According to the study finding, the overall prevalence rate of neonatal sepsis among neonates admitted to NICU was 39.5% (95% CI: 33.7–45.9). Of this, early-onset sepsis was found at 30.2%, whereas late-onset sepsis was 9.3%. Having maternal fever during labor, being unmarried, and prolonged rupture of the membrane were significant independent predictors of outcome variables.

The overall prevalence rate of neonatal sepsis among neonates was 39.5% (95% CI: 33.7–45.9). These values were much higher than those reported in previous studies conducted in Mexico, 4.3% (10); Uganda, 11% (27); Tanzania, 31% (17); India, 32% (36); Wolaita Sodo, 33.8% (37);, Northwest Ethiopia, 34% (38); and Arsi University Teaching Hospital, 34% (22). The possible justification for this discrepancy might have been attributed to the different levels of readiness in following up to prevent infection sources of neonatal sepsis in the pregnancy period, during delivery, and after delivery of maternal and neonatal care. Furthermore, the discrepancy might have been due to methodological and diagnostic parameter differences in confirming neonatal sepsis since our study only focused on the clinical parameters. The study finding is in line with a study conducted in Egypt, 40.7% (29) and Tanzania, about 39% (39). The possible reason might be the similarity in study setup and approach to screening sepsis.

Conversely, studies conducted in different parts of Ethiopia, Shashamane, 78% (23); Arba Minch, 78% (21); Mekelle, 77% (30); Bishoftu, 72.2% (40); Gondar, 65% (24); Jimma, 53% (3); eastern Ethiopia, 46% (26); and Northeast Ethiopia, 79% (20), have much higher prevalence rates compared with the current study. The possible reason for this discrepancy might have been due to the difference in sample size and the socio-demographic and economic status of the study population. Furthermore, different quality healthcare services and accessibility of health facilities would also result in this discrepancy.

Neonates born from unmarried mothers have 18 times increased odds of developing neonatal sepsis compared with their counterparts. This finding is in line with a study conducted in Kenya (18). The possible reason might have been due to the low socio-economic status of unmarried women living in low- and middle-income countries. Moreover, poor maternal care for their new babies might have also contributed to neonatal sepsis.

The odds of developing neonatal sepsis were fivefold higher among neonates born to mothers who had a history of fever during pregnancy as compared with their counterparts. This finding is similar to that of studies conducted in India (8), Ghana (28), and Gondar, Mekelle, and Oromia, Ethiopia (24, 30, 25). This could be because maternal fever during delivery is indicative of maternal infections commonly transmitted from the mother to the baby *in utero* or during passage through the canal, which usually causes early-onset neonatal sepsis.

This study also revealed that mothers who had a history of premature rupture of the membrane are significantly associated with developing neonatal sepsis. The odds of developing neonatal sepsis among neonates born from mothers with a history of premature rupture of the membrane were 7.5 times more likely. Other studies with similar findings were reported in Northeast, eastern, and Mekelle, Ethiopia (20, 26, 30). Early rupture of the membrane increases the chance of microorganisms to ascend from the birth canal into the amniotic sac and fetal compromise as well as asphyxia, which may lead to sepsis.

This study has several limitations. The major limitation is certainly the use of a cross-sectional study design. This study did not include baseline information, which was used to estimate maternal and neonatal characteristics. We did not evaluate the effect of variables related to laboratory investigation due to the lack of accessibility.

### Conclusion and recommendation

This study showed the high prevalence rate of neonatal sepsis among neonates admitted in the neonatal intensive care unit compared with other studies that used similar parameters to diagnose sepsis. Unmarried status of mothers, maternal history of fever during labor, and premature rupture of the membrane were independent risk factors for the development of neonatal sepsis. Therefore, providing training for infection prevention to healthcare professionals is a key element in the reduction of neonatal sepsis. It is better to focus on the identified risk factors in awareness creation in the community.

Therefore, service delivery protocols and standards should be considered for those women at risk (PROM, mothers with elevated temperatures in labor, and others) of developing sepsis.

## Data Availability

The raw data supporting the conclusions of this article will be made available by the authors, without undue reservation.
